# Angiogenesis and Anti-Angiogenic Therapy in Gastric Cancer

**DOI:** 10.3390/ijms19010043

**Published:** 2017-12-23

**Authors:** Henrik Nienhüser, Thomas Schmidt

**Affiliations:** Department of General, Visceral and Transplantation Surgery, University of Heidelberg, Im Neuenheimer Feld 110, 69120 Heidelberg, Germany; Henrik.Nienhueser@med.uni-heidelberg.de

**Keywords:** gastric cancer, angiogenesis, vascular endothelial growth factor, perioperative chemotherapy

## Abstract

Gastric cancer is one of the most frequent malignancies worldwide. Despite improvements in diagnosis and therapy, the overall prognosis remains poor. In the last decade, several anti-angiogenic drugs for cancer treatment have been approved and lately also introduced to gastric cancer treatment. While the initial trials focused only on unresectable or metastatic cancer, anti-angiogenic treatment is now also investigated in the perioperative and neoadjuvant setting. In this review, an overview of the role of angiogenesis and angiogenic factors in gastric cancer as well as anti-angiogenic treatment of gastric cancer is provided. Findings from in vitro and animal studies are summarized and put in a context with translational data on angiogenesis in gastric cancer. The most important angiogenic factors and their effect in gastric cancer are highlighted and clinical trials including anti-angiogenic drugs are discussed. Finally, an outlook of biomarkers for predicting response to anti-angiogenic treatment is presented, the ongoing trials on this topic are discussed and current challenges of anti-angiogenic therapy are outlined.

## 1. Introduction

Gastric cancer is killing more than one million people worldwide every year [1]. Even if diagnosis and perioperative therapy have improved over the last decades, outcome is still poor with overall 5-year survival rates of less than 40% [2,3,4,5]. While the early stages of gastric cancer can be resected in a curative intention, in the case of advanced metastatic tumor stage or unresectability, therapeutic options are limited. Because of low response rates and the development of chemoresistance to the established chemotherapeutic regimens, anti-angiogenic drugs have gained more and more interest in the therapy of gastric cancer [6,7,8].

Blood vessels themselves form an extensive network to nurture all tissues of the body and to supply oxygen. Blood vessels are lined with an inner layer of endothelial cells. Angiogenesis describes the physiological process of the formation of new blood vessels from preexisting ones. While angiogenesis still occurs after birth during organ growth, in the adult, blood vessels are normally quiescent and angiogenesis only occurs physiologically in the cycling ovary and in the placenta during pregnancy [9]. Even though normally quiescent, ECs can sense angiogenic signals and can respond to angiogenic signals by retaining a high plasticity. This is of most importance in pathological conditions such as wound healing and inflammation, however in many disease conditions angiogenesis becomes deregulated and further supports the disease development instead of limiting it [10].

In 1971, Folkman et al. proposed the hypothesis that tumor growth is angiogenesis-dependent [11], which was subsequently proven and is one of the hallmarks of cancer [12]. Since then, several pro- and antiangiogenic factors have been discovered and angiogenic inhibition showed success in the treatment of numerous types of cancer [9,13]. Positive effects by inhibition of angiogenesis were shown among others in breast [14,15,16], lung [17,18], colorectal [19] and gastric cancer [20].

The purpose of this review is to focus on the current data of anti-angiogenic treatment in gastric cancer. We will provide an overview of different angiogenic cytokines and their role in tumor angiogenesis as well as on clinical trials investigating the effect of anti-angiogenic treatment. In the last section, we will give an outlook on the prediction of potential biomarkers and their help for an early diagnosis of the disease.

## 2. Angiogenic Signaling and Pathways

The first described cytokine contributing to tumor angiogenesis was vascular endothelial growth factor (VEGF-A), initially described as VPN (vascular permeability factor), which was discovered in 1983 [21] and fully sequenced in 1989 [22,23]. Even though angiogenesis is a highly complex process, VEGF-A is its central player and is the primary survival factor of vascular endothelial cells (ECs), stimulates proliferation and migration, inhibits apoptosis and modulates their permeability [24]. VEGF-A belongs to a family of cytokines including VEGF-B, -C, -D, -E and placental growth factor (PlGF) [25,26]. Their intracellular signaling is mediated by binding to receptor tyrosine kinases (VEGFR-1, -2, -3) with different affinities. VEGF binds to VEGFR-1 and VEGFR-2, PlGF and VEGF-B bind to VEGFR-1, and VEGF-C and VEGF-D bind to VEGFR-3 and—depending on the species and with a lower affinity—to VEGFR-2 [27]. The main effects on angiogenesis are mediated by VEGFR-2 while signaling via VEGFR-1 and -3 is more complex. The neuropilins NRP1 and NRP2 are co-receptors and enhance the signaling of VEGFR-2 while also signaling by themselves [28,29]. PlGF is another member of the VEGF-family and was first described in the placenta, where it controls trophoblast growth and differentiation [30,31]. While loss of a single allele of VEGF-A is embryonically lethal, PlGF is dispensable and seems to only play a role under disease conditions [32]. In the last decade, it gained more interest since PlGF signaling seems to be involved in the angiogenic process of several solid tumors and leukemia [30,33,34,35].

After identifying the VEGF-family, many other cytokines have been described to regulate the process of angiogenesis in tumors. The angiopoetin/Tie-cascade is one of the most prominent ones. Four different angiopoetins (Ang-1, 2, 3, 4) have been described, all of them binding to the tyrosine kinase receptor tie-2 [36,37] while the exact role of Tie-1, even though it is a co-recpetor of Tie-2, remains unclear. Angiopoetins are critical for vessel maturation and mediate migration, adhesion and survival of endothelial cells. Ang-2 disrupts the connection between the endothelium and perivascular cells and promotes cell death and vascular regression and therefore plays a crucial role in tumor angiogenesis [38]. Another important regulator of angiogenic factors is the fibroblast growth factors (FGF). These cytokines act via binding to four different receptors (FGFR 1–4) and regulate several cell functions such as cell proliferation, migration, survival [39]. Angiogenesis is mainly controlled by FGF-1 and FGF-2 by the activation of the AKT-pathway [40]. In experimental models of ischemic heart disease, FGF-1/2 were shown to induce angiogenesis by increasing coronary artery branching and density [41,42].

As hypoxia is a main driving force of angiogenesis, it is not surprising that upstream to these cytokines angiogenesis is regulated by hypoxia inducible factor 1 (HIF-1). HIF-1 monitors the cellular response to the oxygen levels in solid tumors. Under hypoxia conditions, HIF-1α protein is stabilized and forms a heterodimer with the HIF-1β subunit [43,44]. This complex activates the transcription of numerous target genes in order to adapt the hypoxic environment in human cancer cells [45]. The HIF-mediated adaptive response is orchestrated by HIF prolyl hydroxylase domain-containing enzymes (PHDs), which fulfill specific functions in multiple physiological and pathophysiological processes [46]. In addition to HIF-1, recently HIF-2 gained more interest in the regulation of angiogenesis. Initially, both isoforms were supposed to have similar functions but several publications showed that regulation of erythropoietin (EPO) is mainly regulated by HIF-2 [47].

Beside this typical activator of angiogenesis in gastric cancer, recent studies showed that the non-classical activator tryptase can stimulate angiogenesis in vitro and in vivo. Tryptase is stimulating proliferation of endothelial cells by activation of the proteinase-activated receptor-2 (PAR-2) [48] and VEGF is produced by this process [49]. Tryptase is mainly released by infiltrating mast cells and is thereby marking them as a potential target for the anti-angiogenic therapy in gastric cancer patients [50].

In addition to these pathways, tumor angiogenesis is regulated by abundant other signal cascades and is much more complex. Other pathways such as notch- and wnt-signaling [51,52] regulate the process of angiogenesis in many ways and the interaction between tumor and stroma tissue is especially influenced by other molecules such as integrins [53]. Since this review will focus on the therapeutic implications of angiogenesis inhibition in gastric cancer, it will only highlight the most common pathways.

## 3. Inhibition of Angiogenesis in Preclinical Models

In this section, we will focus on the interaction between gastric cancer cells and angiogenic signaling in vitro and in animal studies.

### 3.1. Experimental In Vitro Data

**Figure 1:**
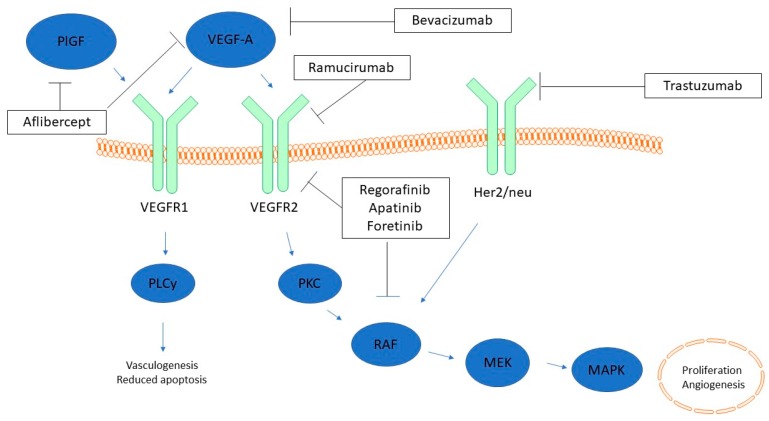
VEGF receptor signaling and targets of anti-angiogenic therapy (modified). PlGF and VEGF-A act through VEGFR1 while VEGFR2 is only stimulated by VEGF-A. Inhibitors of the pathways are shown in boxes. Downstream pathways are simplified for better orientation. PlGF = Placental growth factor; VEGF = Vascular endothelial growth factor; VEGFR = Vascular endothelial growth factor receptor; Her2/neu = human epidermal growth factor receptor 2; PLCy = Phospolipase C y; PKC = protein kinase C; RAF = Rapidly accelerated sarcoma kinase; MEK (MAPKK) = Mitogen-acitvated protein kinase kinase; MAPK = mitogen-activated protein kinase. Blue arrow = activation; black T-bar arrow = inhibition.

Several studies on blocking angiogenic pathways in gastric cancer cell lines have been reported. The first monoclonal antibody against VEGF was bevacizumab (avastin) which has been shown to inhibit angiogenesis in several solid tumors [19]. Treatment of gastric cancer cell lines with bevacizumab showed decreased cell growth and increased apoptosis rates [54]. Besides inhibition by blocking VEGF via bevacizumab, there are two inhibitors of the VEGFR-2, the monoclonal antibody ramucirumab and the tyrosine kinase inhibitor apatinib. Both were shown to inhibit cell growth in vitro [55]. Silencing VEGF by specific siRNA led to the same effects with decreased proliferation rates and reduced cell cycling [56]. One possible explanation for this is the activation of proliferation-mediating signaling pathways, such as PI3K/Akt, by secreted VEGF [57]. Human umbilical vein endothelial cells (HUVECs) as a model of angiogenesis showed upregulated mRNA levels of VEGFR-1/-2 and VEGF when co-cultured with gastric cancer cells in vitro [58]. A summary of the different drugs and their target in the angiogenic signaling is provided in Figure 1.

All these data suggest that gastric cancer cells have a high angiogenic potential by secreting angiogenic cytokines both to stimulate endothelial cells as well as in an autocrine loop to support their own growth. Altogether, migration and proliferation in gastric cancer cells are highly regulated by the VEGF pathway.

While there is abundant data on the inhibition of VEGF-mediated pathways in gastric cancer, little is known about the effect of blocking PlGF in gastric cancer cell lines. Akrami et al. reported an increased apoptosis rate and decreased migration in AGS cells by knocking down of PlGF [59]. The same group reported less tumorigenicity in AGS and MKN-45 cells after knockdown of PlGF [60]. In this study, Mahmoodi et al. assessed stem cell properties in these cell lines and found less self-renewal capacity, decreased matrix metalloprotease activity and decreased transcription activity [60]. Inhibition of Angiopoetin-1 in vitro shows similar effects with reduced cell proliferation and cell cycle activity [61,62,63]. Downregulation of Angiopoetin-2 led to similar results with decreased proliferation rates in gastric cancer cell lines [64]. For PlGF, no studies are available on the effect of an antibody-mediated blockage and for a better understanding of PlGF-mediated angiogenesis in gastric cancer this pathway needs to be further elucidated.

All in all, blocking of VEGF, Angiopoetin-1/2 (and PlGF) was found to have inhibitory effects on cell proliferation and migration. Beside these expected effects, there are several studies investigating the interaction between anti-angiogenic therapy and chemoresistance. Induction of angiogenesis has been shown to upregulate β-tubulines which contribute to chemoresistance in breast cancer [65,66]. Blocking this pathway leads to increased chemosensitivity of gastric cancer cells towards paclitaxel [67]. Zhao et al. reported inducing multidrug resistance by the upregulation of HIF [68]. One reason for this enhanced sensitivity might be decreased apoptosis resistance via modulation of p53 and NF-kB [69] and higher expression levels of bcl-2 as well as lower expression of bax [70]. In summary, inhibition of angiogenesis does not only inhibit cell migration and proliferation in a direct way but also seems to enhance the effect of chemotherapy. This effect might be due to vascular normalization promoted by anti-angiogenic therapy. Compared to normal blood vessels, tumor vessels are structurally and functionally abnormal and impairing the delivery of chemotherapeutic agents [71]. Jain proposes that, by anti-angiogenic treatment, the tumor vessels normalize and thereby reestablish a proper blood flow. By this, the chemotherapeutic agents can reach therapeutic concentrations and the addition of anti-angiogenic treatment to chemotherapy has an adjuvant effect.

### 3.2. Experimental In Vivo Data

Most of the effects which are shown in vitro were confirmed in animal models. Blocking the effects of VEGF by silencing RNA in gastric cancer cell lines led to reduced tumor volume after implantation into nude mice [72]. The same effect was observed when mice were treated with apatinib after tumor implantation [55]. In mouse models simulating peritoneal metastasis, administration of bevacizumab significantly decreased tumor volume and the amount of malignant ascites [73,74]. One important effector in this signaling cascade is the STAT3-pathway. This transcription factor regulates cell growth, migration and angiogenesis in multiple ways. Inhibition of STAT3 leads to significant lower expression of VEGF and reduced tumor progression after implantation of gastric cancer cells in nude mice [75]. The effect of STAT3 is inhibited by GP130, which acts as a binding site for IL-6. Inactivation of this negative inhibition in GP130^F/F^ mice leads to rapid tumor development in the epithelium of the glandular stomach [76]. This animal model shows that the angiogenic and oncogenic pathways are linked to each other and tumorigenesis of gastric cancer is closely related to the activation of angiogenic pathways.

This relation is also observable when using combination therapy against angiogenic and proliferative pathways. The human epidermal growth factor receptor (Her2/neu) regulates cell growth and proliferation via RAS-MAP-kinase signaling. Inhibition of this receptor by the antibody trastuzumab was shown to improve survival in breast cancer patients [77]. After implanting gastric cancer cells into nude mice, a combined inhibition of VEGF and epidermal growth factor receptor led to significantly decreased tumor growth [78]. Trapping VEGF in addition to Her2/neu blockage showed a significant decrease in tumor proliferation and increase in apoptosis of tumor cells compared to each of the agents alone [79]. The fusion protein aflibercept traps VEGF and PlGF in vivo and is currently being investigated in a clinical trial (NCT01747551) in addition to standard chemotherapy in gastric cancer patients.

Beside this VEGF-specific inhibition, the effect of HIF-1 blockage has been investigated in animal models in several ways. Treatment of subcutaneous xenografts with an inhibitory HIF-1 compound results in smaller and less vascularized tumors after implantation into nude mice [80]. In addition, human gastric cancer cells expressing a dominant negative form of HIF-1 showed slower tumor growth, smaller overall vessel area and hampered vessel maturation when implanted orthotopically in nude mice [81]. While these effects have been shown in many other types of cancer [82], the interaction of HIF-1 with the gastric microbiome might have an organ-specific influence. Chronic Infection with *H. pylori* is a driving factor for the development of reactive oxygen species (ROS) due to neutrophil infiltration in response to *H. pylori*. This chronic infection causes epithelial cell injury and progressive DNA damage [83]. There is evidence that gastric epithelial ROS may lead to HIF-1α expression under normal oxygen conditions [84] and facilitate the tumor angiogenesis of gastric cancer. Non-steroid antiphlogistics (NSAIDs) have been shown to protect from gastric cancer. In a rat model, COX-inhibition by NSAIDs showed impaired tumor angiogenesis, and decreased HIF-1 levels were observed in cells after exposure to NSAIDs.

## 4. Translational Data and Angiogenic Factors as Biomarkers

These findings from in vitro and animal studies were investigated in patients and most of these molecules have been shown to be predictive in the evaluation of the response and prognosis of gastric cancer. High serum levels of VEGF-A were shown to correlate with advanced tumor stages and decreased survival [85,86]. Wang et al. [87] found higher levels of VEGF-C in patients with lymph node metastasis. Overexpression of Angiopoetin in serum and tumor tissue is associated with poor survival. A combination of both factors by calculating a Angiopoetin/VEGF-ratio is an independent predictor for clinical and histopathological response to chemotherapy in gastric cancer but not esophageal cancer [88,89,90]. While for colorectal and pancreatic cancer compartment-specific expression, data of angiogenic molecules is available [91,92], the exact expression pattern of angiogenic molecules in gastric cancer and its microenvironment is still not clear. Based on the data from the AVAGAST trial (see below), Hacker et al. [93] found that the baseline plasma level of Angiopoetin-2 is an independent predictor of overall survival and correlated with the frequency of liver metastasis. Baseline plasma levels of Ang-2 were significantly lower in Asian patients and did not predict the response to the therapy with Bevacizumab. The study population was divided into the two subgroups Asian-Pacific and Non-Asian patients. In the non-Asian subgroup, van Cutsem et al. [94] found high levels of VEGF-A and low levels of neuropilin-1 associated with a trend towards improved overall survival. Similar to the findings by Hacker et al., all these effects were only present in non-Asian patients while in Asian patients no significant association between the described biomarkers and survival was observed. In contrast to these international studies, a smaller study, only including Asian patients, did find a significant association between VEGF-A, Ang-3, Neuropilin and HIF-1 and advanced tumor stage as well as poor survival [95]. A summary of all outcome-related factors is provided in Table 1.

Beside predicting the response to anti-angiogenic therapy by cytokine plasma levels, several clinical symptoms have been shown to be significantly associated with response to anti-angiogenic therapy. The development of hypertension under a therapy with bevacizumab predicted overall survival in colorectal cancer patients [106,107] and proteinuria was associated with [108] survival as well. As the grade of proteinuria is highly dependent on the combination with other chemotherapeutic drugs, the establishment as a marker for monitoring response to anti-angiogenic therapy might be problematic.

The reason for the development of hypertension is not completely understood and was reviewed by several other authors [109,110]. One explanation discussed by these authors is the impaired NO-production from the endothelial cells by inhibition of VEGF-signaling. In the end, it remains unclear which unknown interactions lead to the development of hypertension in patients with anti-angiogenic treatment. As patients usually receive a combination therapy of anti-angiogenic drugs with chemotherapeutic agents, it is hard to define what specific interaction is causing the hypertension.

In contrast to the studies that showed a correlation between plasma levels of VEGF and response to anti-angiogenic therapy, other authors did not see this effect in other types of cancer. In four phase III studies in patients with colorectal, renal and lung cancer, the pretreatment levels of VEGF-A were associated with the overall survival but did not predict response to the therapy with bevacizumab [111], indicating a prognostic rather than a predictive value of circulation VEGF.

As initially described, the role of tryptase releasing mast cells in gastric cancer angiogenesis has been recently investigated. A higher density of mast cells was shown to be associated with a higher general vascularized area [50] in primary tumors and lymph node metastasis from patients undergoing resection of gastric cancer. In what way this parameter is helpful to predict survival and response to anti-angiogenic therapy has to be shown by clinical trials in the future.

All in all, the response to anti-angiogenic treatment might be associated with biomarkers but monitoring only specific markers seems to be insufficient and prospective clinical trials investigating this topic are needed [112].

## 5. Clinical Trials

Because of this striking preclinical data, inhibition of angiogenesis in gastric cancer was transferred to clinical use. The first trial showing positive effects by inhibition of angiogenesis was the REGARD trial conducted between 2009 and 2012 [20]. Patients with previously treated advanced gastric cancer were either treated with ramucirumab, a VEGFR-2 antibody, or with a placebo. Patients who received ramucirumab had significantly prolonged overall survival of 5.2 months compared to 3.8 months and progression-free survival of 2.1 months compared to 1.3 months in the control group. An additional study, the RAINBOW trial, was conducted between 2010 and 2012 and showed an increased overall survival for patients treated with ramucirumab in combination with paclitaxel compared to patients treated only with paclitaxel. The survival benefit in this study was 2.2 months and the progression-free survival benefit was 1.5 months [113]. Based on these two studies, ramucirumab was approved by the FDA in 2014 for the second-line treatment of advanced gastric cancer. Comparing treatment with ramucirumab with standard therapy, the incidence of side effects is comparable with only significantly more treatment-related hypertension in the patients receiving ramucirumab. Treatment-related death occurred in 2% of the patients in the REGARD trial and showed no difference to the patients receiving standard therapy. An overview of the phase-III trials with anti-angiogenic drugs is presented in Table 2.

Before this approval, the AVAGAST and AVATAR trials, comparing the VEGF-antibody bevacizumab plus cisplatin/capecitabine to chemotherapy alone in different populations, failed to show any benefit in overall survival [114,115]. Patients treated with bevacizumab were shown to have significantly longer progression-free survival and higher response rates to chemotherapy but did not have any benefit in terms of overall survival. Subgroup analysis of patients in the AVAGAST trial by region showed that patients in North and South America seem to benefit from an anti-angiogenic therapy (overall survival of 11.5 months compared to 6.8 months in the control group) while this effect was not seen in Asian patients (13.9 months compared to 12.9 months). European patients showed intermediate results (11.1 months compared to 9.6 months). This missing effect was confirmed by the AVATAR trial which showed no benefit of bevacizumab in a Chinese patient population. However, the explanation for the different response to anti-angiogenic treatment among the different genetic backgrounds remains unclear.

Ma et al. [118] investigated bevacizumab plus the standard chemotherapy protocol DOF (docetaxel/oxaliplatin/5-FU) compared to chemotherapy alone in a neoadjuvant setting. Patients who received bevacizumab had longer progression-free survival and higher rates of complete surgical resection but did not show any benefit in overall survival. Cunningham et al. [117] reported similar survival rates but impaired wound healing and an increased rate of anastomotic leakage in patients undergoing preoperative treatment with bevacizumab. Taken together, bevacizumab, for some reason, seems to have no positive effects on overall survival compared to ramucirumab, even if it is blocking the same pathway. Because of this lack of clinical evidence and its negative effects on wound healing, bevacizumab is not approved for the treatment of advanced gastric carcinoma.

After the positive results of the REGARD and RAINBOW trial, a large number of drugs were tested for angiogenic inhibition in gastric cancer. The small-molecule inhibitor of VEGFR-2 Apatinib was tested in patients with previously treated, advanced gastric carcinoma and showed prolonged overall and progression-free survival [116]. The multi kinase inhibitor regorafinib, that targets angiogenic (VEGFR-1 and -2, tie-2), stromal (PDGF-β) and oncogenic (RAF, RET and Kit) kinases was tested in patients with advanced gastric carcinoma [119]. This phase-II trial showed prolonged progression-free survival compared to patients treated with a placebo. Survival data will be expected from the ongoing phase-III study. Similar results were obtained by testing the multi-kinase inhibitor foretinib which has an inhibitory effect on VEGFR2 and Tie-2. This treatment did not show any benefit in an unselected patient cohort with advanced gastric cancer [120].

A systematic review on this topic by Shan et al. [121] found a total number of 16 trials investigating tyrosine kinase inhibitors in the therapy of gastric cancer. Only apatinib and, to a certain extent, regorafenib, showed positive results while all other therapies failed to show any benefit compared to standard therapy. A limitation of clinical trials is the different response to anti-angiogenic therapy among the different ethnicities. Subgroup analysis of the AVAGAST trial showed that non-Asian patients more likely benefit from an anti-angiogenic therapy than Asian patients. In the overall study population, this effect was not observed. Further trials investigating the effect of ethnicity on the response are needed to answer this question.

Nevertheless, the angiogenic phenotype of gastric cancer and its susceptibility to anti-angiogenic therapy could be confirmed by clinical trials even if most of the anti-angiogenic drugs failed to show a benefit in a clinical setting. In addition, the survival benefit was statistically significant but differences were in the range of months. Anti-angiogenic therapy cannot stop progression of gastric cancer and is only an option to prolong survival time in a certain manner. In advanced gastric cancer, the tumor develops escape strategies and quickly overcomes the inhibition of angiogenic pathways. Because of these limitations, it is crucial to identify biomarkers that are able to predict responses and prognoses related to anti-angiogenic treatment.

## 6. Challenges

Despite the achievements of these anti-angiogenic drugs, there are still a lot of remaining challenges. The survival benefit by inhibition of angiogenesis only leads to short survival benefits and tumors seem to develop escape mechanisms within a short time. Tumors seem to acquire specific abilities to escape the anti-angiogenic therapy, including upregulation of compensatory pathways [122], vasculogenic mimicry [123] and recruitment of bone marrow-derived cells [124,125]. In addition to these escape mechanisms, several authors describe increased invasiveness and metastasis in patients treated with angiogenic inhibitors. Ebos et al. [126] showed that short-term treatment with sunitinib leads to acceleration of metastasis in a mice model, suggesting a “metastatic conditioning” by this treatment. The same findings were reported by Paez-Ribes [127] in a mice model of pancreatic cancer and glioblastoma. Although some other studies did not reproduce this effect [128], the possibility of increasing tumor aggressiveness by anti-angiogenic therapy has to be regarded as a major caveat of treatment, especially in patients with non-metastatic, localized gastric cancer.

This leads to the question of anti-angiogenic treatment as part of pre- or perioperative therapy. In the trial by Cunningham et al. [117], no survival benefit was shown by adding bevacizumab to the standard perioperative chemotherapy. Currently, the RAMSES/FLOT7 trial (NCT02661971) is recruiting patients with resectable gastric or GE junction carcinoma to investigate the effect of adding ramucirumab to the standard perioperative chemotherapy regimen (FLOT) and initial results are expected in early 2019. Regarding the safety of anti-angiogenic therapy in perioperative treatment, the STO3 trial did show a significant increase in wound healing complications and an increased rate of anastomotic leakage (24% vs. 10%) in patients treated with bevacizumab (REF). Because of these findings, patients with lower esophageal or GE junction carcinoma were excluded from the recruitment. It remains to be seen whether the RAMSES trial will show similar results or if the wound healing complications will be less in ramucirumab-treated patients compared with those receiving bevacizumab. The implication of anti-angiogenic therapies in perioperative treatment of gastric cancer will only be reasonable if surgical complications are comparable to standard chemotherapy.

Besides the challenges posed by the perioperative treatment of metastatic disease, the main challenge is patient selection. In all the described trials, no assessment of the baseline expression of the angiogenic factor or mutational status was performed before the treatment. This might be one reason why, in these unselected patient cohorts, most of the anti-angiogenic agents failed to show a general survival benefit. As mentioned above, the geographical background of the patients seems to have an important influence on the effectiveness of anti-angiogenic treatment. While European and American patients seem to benefit in some way, most Asian patients failed to show any benefit compared to standard therapy.

With the currently available biomarkers, no appropriate prediction of either response or outcome of a treatment with anti-angiogenic drugs, seems possible. Prospective randomized studies will be needed to elucidate this field and make possible better patient selection for the available drugs.

## Figures and Tables

**Table 1:** Potential biomarkers predicting survival and metastasis in gastric cancer.

Angiogenic Factor	Detection	Lymph Nodes	Distant Metastasis	Survival
VEGF-A VEGF-C	VEGF-A is elevated in gastric cancer patients [96,97,98,99]	VEGF-A and VEGF-C is elevated in patients with lymph node metastasis [87]	VEGF-A is elevated in patients with distant metastasis [87,89,97]	Elevated VEGF-A is associated with worse prognosis [87,100,101]
Angiopoetin-1/2	Ang-2 is elevated in gastric cancer patients [102,103]	Ang-2 is elevated in patients with lymph node metastasis [103]	Ang-2 is elevated in patients with liver metastasis [93]	Higher levels are correlated with advanced stages [93]
Neuropilin-1/2	No clear evidence of correlation	No clear evidence of correlation	No clear evidence of correlation	Low levels are associated with shorter survival [94]
PlGF	Higher expression of PlGF in tumor tissue compared to normal mucosa [104]	No clear evidence of correlation	No clear evidence of correlation	No correlation with survival as a single factor [105]

**Table 2:** Overview of phase-III studies in gastric cancer including anti-angiogenic therapy.

Study	Patients	Region of Recruitment	Treatment	Previous Therapy	Median Overall Survival	Progression-Free Survival
Ohtsu et al. (AVAGAST) 2011 [114]	*n* = 774 Unresectable locally advanced/metastatic gastric cancer	Asia-Pacific region: 49% Europe: 32% Pan-America: 19%	Bevacizumab + Fluoropyrimidin/Cisplatin vs. Placebo + Fluoropyrimidin/Cisplatin	1st line	12.1 vs. 10.8 months (*p* = 0.1)	6.7 vs. 5.3 months (*p* = 0.0037)
Shen et al. [AVATAR] 2015 [115]	*n* = 202 Unresectable locally advanced/metastatic gastric cancer	China: 100%	Bevacizumab + Capecitabine/Cisplatin vs. Placebo + Capecitabine/Cisplatin	1st line	10.5 vs. 11.4 months (*p* = 0.56)	6.3 vs. 6.0 months (*p* = 0.47)
Fuchs et al. [REGARD] 2014 [20]	*n* = 355 Unresectable or metastastic, locally recurrent gastric or GE junction adenocarcinoma	North America, Europe, Australia: 69% Asia: 8% South/Central America, India, Middle East: 23%	Ramucirumab vs. Placebo	2nd line	5.2 vs. 3.8 months (*p* = 0.047)	2.1 vs. 1.3 months (*p* = 0.001)
Wilke et al. [RAINBOW] 2014 [113]	*n* = 665 Unresectable or metastastic gastric or GE junction adenocarcinoma	Europe, Australia, USA: 60% South/Central America: 7% Asia: 33%	Ramucirumab + Paclitaxel vs. Placebo + Paclitaxel	2nd line	9.6 vs. 7.4 months (*p* = 0.017)	4.4 vs. 2.9 months (*p* < 0.0001)
Li et al. 2016 [116]	*n* = 267 advanced or metastatic gastric or gastroesophageal junction adenocarcinoma	Asia: 100%	Apatinib vs. Placebo	3rd line	6.7 vs. 4.9 months (*p* = 0.149)	2.6 vs. 1.8 months (*p* = 0.001)
Cunningham et al. [ST03] 2017 [117]	*n* = 1063 Resectable adenocarcinoma of the stomach/GE junction/lower esophageus	Europe: 100%	Bevacizumab + epirubicine/capecitabine/cisplatin vs. Placebo + epirubicine/capecitabine/cisplatin	perioperative	3-year-OS:* 48.1 vs. 50.3% (*p* = 0.36)	No concrete time reported: HR: 1.05 (95% CI: 0.89–1.23); *p* = 0.56

* Highlighted, as it is different than the median over survival as stated in the header of the column.
